# Primary gastric choriocarcinoma: A case report

**DOI:** 10.3389/fsurg.2022.1009119

**Published:** 2022-11-02

**Authors:** Zhang Xusheng, Yan Yuke, Meng Yun, Guo Huijun, Peng Jiangshan, Du Xueqin, Yang Xiaojun

**Affiliations:** ^1^ The 1st Clinical Medicine College, Gansu University of Chinese Medicine, Lanzhou, China; ^2^ Department of General Surgery, Gansu Provincial Hospital, Lanzhou, China; ^3^ School of People's Clinical Medicine, Lanzhou University, Lanzhou, China; ^4^ Gansu Key Laboratory of Molecular Diagnostics and Precision Medicine for Surgical Oncology, Gansu Provincial Hospital, Lanzhou, China; ^5^ Gansu Research Center of Prevention and Control Project for Digestive Oncology, Gansu Provincial Hospital, Lanzhou, China; ^6^ College of Clinical Medicine, Ningxia Medical University, Yinchuan, China

**Keywords:** choriocarcinoma, primary gastric choriocarcinoma, pathology, treatment, case report

## Abstract

**Background:**

Choriocarcinoma is a malignant tumour of trophoblastic origin. Most are gestational choriocarcinomas, which usually occur in women with an epithelial origin of the placental chorionic villi and are associated with pregnancy. It mainly originates in the gonads such as the ovaries and testes. However, it rarely occurs in the stomach and is known as primary choriocarcinoma (PGC).

**Case presentation:**

A 69-year-old man complained of abdominal distention for 3 years, which worsened 1 week later. Gastroscopy showed chronic atrophic gastritis C1 (C1: indicates atrophic gastritis involving the sinus region); the pathology report of the gastroscopic specimen showed high-grade epithelial tumours in the mucosal glands. We diagnosed an occupying lesion in the stomach and performed a laparoscopically assisted distal gastrectomy and Billroth type 1 anastomosis. Postoperative pathology showed “gastric choriocarcinoma with cancerous tissue invading the entire gastric wall”. The patient was discharged on the 11th postoperative day as there were no postoperative complications. The patient was followed up until June 2022 with a good recovery and no recurrence.

**Conclusion:**

We encountered a case of Primary Gastric Choriocarcinoma, where the cancerous tissue invades the full thickness of the gastric wall.

## Background

Choriocarcinoma is a malignant tumour of trophoblastic origin (1). The vast majority of choriocarcinomas are associated with pregnancy, most commonly in the uterus, and originate mainly in the ovaries, testes and other gonads. However, primary gastric choriocarcinoma is is a rare malignancy, accounting for approximately 1% of gastric cancers (2, 3). Here, we report a case of primary gastric choriocarcinoma in which cancerous tissue invaded the entire gastric wall.

## Case report

**Figure 1:**
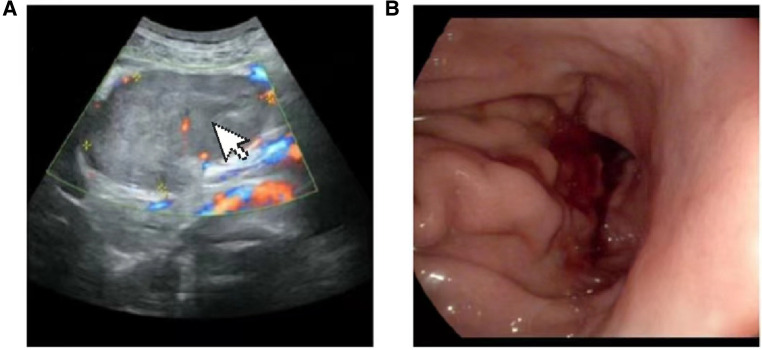
(**A**) Doppler ultrasound:upper abdominal solid mass. (**B**) Gastroscopy: chronic atrophic gastritis C1: gastric antrum mass (to be examined).

**Figure 2:**
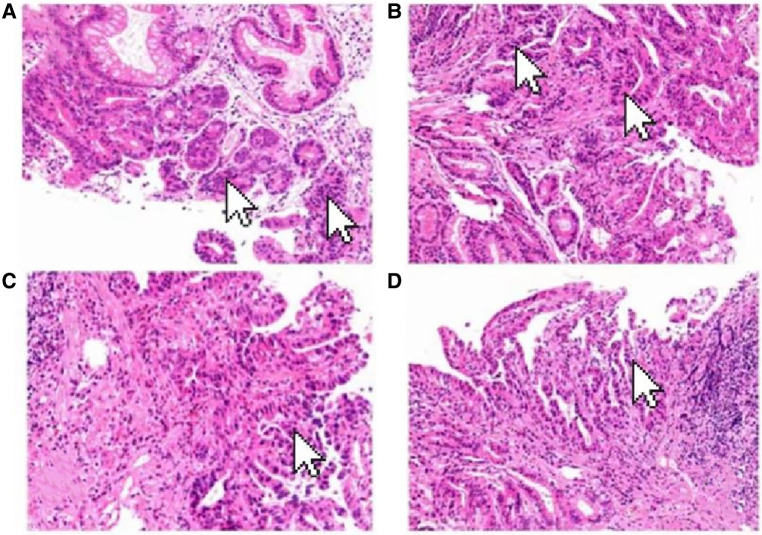
Pathological examination of the specimen taken under gastroscopy revealed high-grade intraepithelial neoplasia in the (sinus) mucosal glands (arrow). (HE×100).

**Figure 3:**
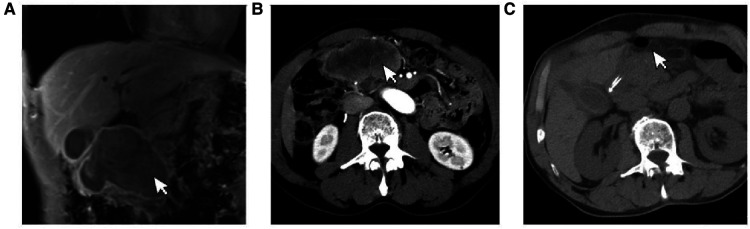
(**A**) The gastric antrum occupies space, the main lesion is located outside the gastric wall. (**B**) Patient's preoperative CT showed obvious lesions. (**C**) Postoperative CT of the patient showed significant resection of the lesion.

**Figure 4:**
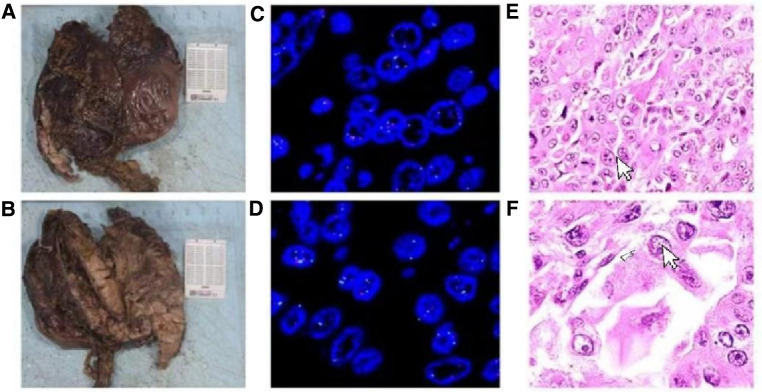
(**A,B**) Specimen: the size of the mass is about 9 cm × 7 cm × 6 cm, and the surface of the slice is red and gray, indicating solid, soft tissue. (**C-F**) Primary choriocarcinoma of the stomach, with Ovoid or polygonal, with hyaline, lightly stained or granular cytoplasm and a single vacuolated nucleus (HE ×100).

**Figure 5:**
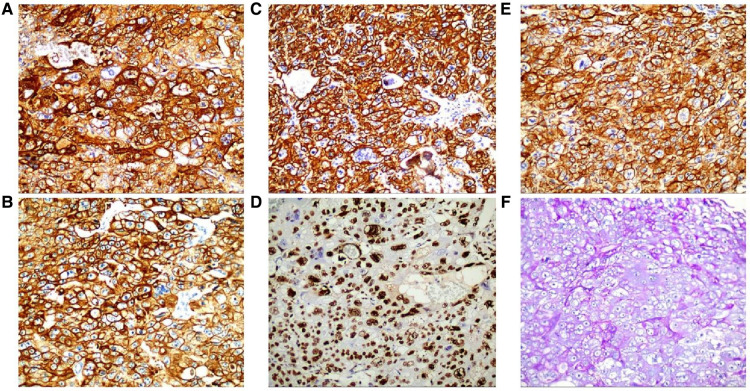
Immunohistochemistry: CD10 (+) (**A**), ck8/18 (+) (**B**), CK19 (+) (**C**), CKP (+) (**D**) and Ki-67 labelling index of 60% (+) (**E**) (HE ×200). Histochemical staining: PAS (+) (**F**) (SP ×200).

**Figure 6:**
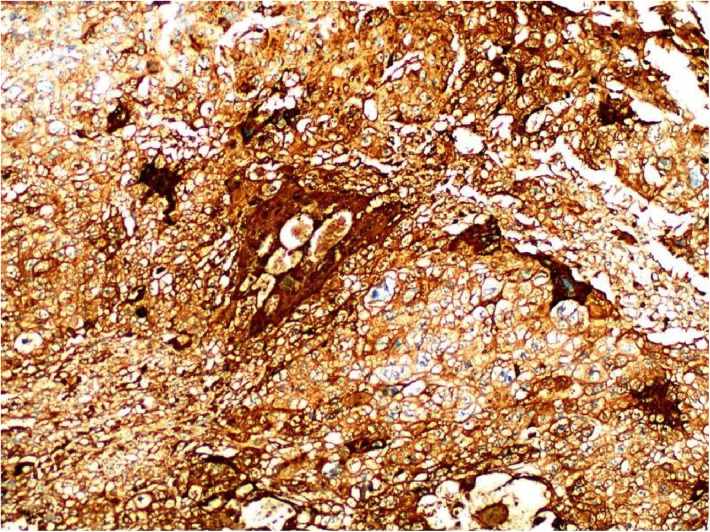
Immunohistochemistry: HCG(+) (SP ×100).

A 69-year-old male presented to our hospital complaining of abdominal distension and discomfort for 3 years, worsening for 1 week. His medical history included chronic appendicitis and he had undergone an appendectomy 2 years ago. Recently, there was no significant change in weight. One week ago, the patient felt a marked increase in abdominal distension and pain, mainly in the upper and middle abdomen, and presented to our hospital. The results of the examination were as follows: normal vital signs, fecal occult blood test (++), ALB: 33.05 g/L, Hb: 96 g/L and HCG-β: 66.12 mIU/ml. Liver and kidney function, electrolytes and blood glucose were normal. Abdominal Doppler ultrasound showed a solid mass in the upper abdomen, which was suspected to be gastric cancer (Figure 1A). Gastroscopy revealed chronic atrophic gastritis stage C1 (C1: indicates atrophic gastritis involving the gastric sinus region) (Figure 1B). Pathological examination of the specimen taken under gastroscopy revealed high-grade intraepithelial neoplasia in the (sinus) mucosal glands (Figures 2A–D). Tumour marker examination showed normal tumour series CEA, AFP and CA199 except CA72-4: 10.07 U/ml (0–6 U/ml). MRI of the abdomen showed that the gastric sinus was occupied, with the main lesion located outside the gastric wall (Figure 3A). Biopsy consultation at another hospital showed a moderate chronic mucosal inflammation with mild intestinal metaplasia. Considering the patient's current relevant investigations and findings, we considered a gastric occupying lesion (suspected mesenchymal tumour). The patient was stable and had clear indications for surgery. We therefore performed a laparoscopic-assisted distal gastrectomy and a Billroth type 1 anastomosis. The postoperative gross specimen showed a mass measuring approximately 9 cm × 7 cm × 6 cm, which was reddish-grey in section, suggestive of solid soft tissue (Figures 4A,B). Pathology of the postoperative specimen showed PGC cancerous tissue invading the entire gastric wall (T4aN0M0). Metastases were found only in the vascular system and no other system had been identified. No tumour was found at either side of the resection margins. Microscopic examination showed marked tumour heterogeneity with visible cancerous areas, tumour cells of variable size, ovoid or polygonal in shape, with clear, lightly stained or granular cytoplasm, a single vacuolated nucleus with a distinct nucleolus, many nuclear fission signs, some cells could appear multinucleated, with abundant cytoplasm and large nuclear heterogeneity. The cells are of cytotrophoblastic and syncytial trophoblastic origin and are devoid of villous structures (Figures 4C–F). Immunohistochemistry showed CD10 (+) (Figure 5A), ck8/18 (+) (Figure 5B), CK19 (+) (Figure 5C), CKP (+) (Figure 5D) and a Ki-67 labelling index of 60% (+) (Figure 5E). Histochemical staining showed PAS (+) (Figure 5F), while immunohistochemistry showed positive for HCG antibodies (Figure 6). A postoperative blood HCG-β (β-human chorionic gonadotropin) test was performed at 66.12 mIU/ml, followed by cranial MRI and chest and abdominal CT, which showed no signs of distant metastases. The diagnosis of PGC was now almost clear. The patient was discharged on the 11th postoperative day and was followed up until June 2022, with good recovery and no recurrence.

## Discussion and conclusions

Primary gastric choriocarcinoma was first described by Davidsohn in 1905 (4) and accounts for approximately 0.8% of all gastric cancers (5). PGC is most commonly seen in older men, with a male to female ratio of 2.3:1 (6). The tumour is mainly located in the gastric sinus, followed by the gastric body and least in the cardia (7, 8). The clinical presentation is similar to that of gastric adenocarcinoma, with only 25% of patients developing choriocarcinoma (9). However, primary gastric choriocarcinoma is more likely to cause gastrointestinal bleeding than other tumours (10). Very few cases of primary gastric choriocarcinoma have been reported in China, most of which are associated with elevated serum β-HCG (human chorionic gonadotropin) levels, nausea, vomiting and gynaecomastia (7). The pathogenesis of the disease is unclear and several theories exist. The most accepted mechanism is Pick's theory of dedifferentiation, which states that primary gastric choriocarcinoma originates from the reverse differentiation of gastric adenocarcinoma. This theory suggests that existing gastric adenocarcinoma cells differentiate into embryonic stages and become trophoblast cells, which in turn differentiate into choriocarcinoma (11). Its diagnosis remains very difficult. The diagnosis of PGC must be made with great caution and the following conditions must be met: firstly, the presence of a tumour in the stomach must be excluded from any other occult primary site; secondly, the morphology of the tumour is similar to that of choriocarcinoma in other sites and the tumour cells express HCG by immunohistochemistry; and finally, HCG is elevated in serum and urine preoperatively but decreases to normal levels after gastrectomy and chemotherapy. Therefore, when preoperative gastroscopy reveals a malignant gastric ulcer, a tissue biopsy is required to improve the preoperative diagnosis. The diagnosis of primary gastric choriocarcinoma is then made postoperatively based on pathological features and immunohistochemical findings. In this case the preoperative gastroscopic pathology showed only high-grade intraepithelial neoplasia of the mucosal glands, yet the postoperative pathology report showed gastric choriocarcinoma. The serum HCG level decreased from 66.12 miu/ml postoperatively to 42.86 miu/ml (one week postoperatively). We then performed various imaging examinations such as cranial MRI and chest CT to exclude other tumours. We eventually combined the imaging findings, postoperative pathological features, immunohistochemical results and serological tests to make a final diagnosis of primary gastric choriocarcinoma. Its histopathology was rapid in most patients with PGC, with a poor prognosis. Most patients survive for 6 months (12) and usually die of blood transmission within the first year of diagnosis. Lymph nodes, liver, peritoneum and lung are common sites of metastasis (5). Poor prognostic factors affecting overall survival are mainly liver metastases, which usually lead to liver failure, residual tumour after surgery, and lack of chemotherapy (13). Due to its combination of gastric and choriocarcinoma characteristics, there is no standard treatment protocol. For early stage patients, postoperative chemotherapy is the treatment of choice after gastrectomy and lymph node dissection (13). For patients with locally advanced disease, systemic chemotherapy can be followed by radical gastrectomy combined with postoperative chemotherapy. For patients with advanced cancer, systemic chemotherapy is primarily used. Previous case reports have described (5) the use of VIP, BEP (bleomycin, etoposide, and cisplatin), EMA/CO (eto-poside, methotrexate, actinomycin D, cyclophosphamide, and vincristine), and fluorouracil plus cisplatin, but their efficacy was poor. However, Ceilesh A et al. (2) found that VIP chemotherapy regimens were effective in the early treatment of metastatic primary gastric choriocarcinoma. Picazo (14) found that two patients who received chemotherapy had a mean survival of 120 days. Monitoring of serum HCG levels may help to assess the effectiveness of treatment and tumour recurrence. As a rare disease, PGC is particularly important to diagnose early and accurately through various tests as its clinical presentation is very similar to that of other tumours. To date, there is no standard treatment for patients with PGC, so it is important to explore standard treatment options. In this case, the postoperative blood HCG-β test was 66.12 mIU/ml: since then, the HCG-β level has been measured regularly (1 week postoperative: 42.86 mIU/ml; 2 months postoperative: 15.56 mIU/ml), showing a gradual decrease and a stable trend, and together with imaging examinations (cranial MRI, chest and abdominal CT), suggesting that there are no signs of recurrence or metastasis, but close follow-up is still needed. The patient should be followed up closely.

## Data Availability

The original contributions presented in the study are included in the article/Supplementary Material, further inquiries can be directed to the corresponding author/s.
